# The potential of autologous regulatory T cell (Treg) therapy to prevent Cardiac Allograft Vasculopathy (CAV) in paediatric heart transplant recipients

**DOI:** 10.3389/fimmu.2024.1444924

**Published:** 2024-09-09

**Authors:** Apoorva Aiyengar, Marco Romano, Michael Burch, Giovanna Lombardi, Giorgia Fanelli

**Affiliations:** ^1^ Department of Cardiology, Great Ormond Street Hospital NHS Foundation Trust, London, United Kingdom; ^2^ Research Department of Children’s Cardiovascular Disease, Institute of Cardiovascular Science, University College London, London, United Kingdom; ^3^ Peter Gorer Department of Immunobiology, School of Immunology and Microbial Sciences, King’s College, London, United Kingdom

**Keywords:** regulatory T cells, transplantation, cell therapy, cardiac allograft vasculopathy (CAV), paediatric, thymic

## Abstract

Paediatric heart transplant is an established treatment for end stage heart failure in children, however patients have to commit to lifelong medical surveillance and adhere to daily immunosuppressants to minimise the risk of rejection. Compliance with immunosuppressants can be burdensome with their toxic side effects and need for frequent blood monitoring especially in children. Though the incidence of early rejection episodes has significantly improved overtime, the long-term allograft health and survival is determined by Cardiac Allograft Vasculopathy (CAV) which affects a vast number of post-transplant patients. Once CAV has set in, there is no medical or surgical treatment to reverse it and graft survival is significantly compromised across all age groups. Current treatment strategies include novel immunosuppressant agents and drugs to lower blood lipid levels to address the underlying immunological pathophysiology and to manage traditional cardiac risk factors. Translational researchers are seeking novel immunological approaches that can lead to permanent acceptance of the allograft such as using regulatory T cell (Tregs) immunotherapy. Clinical trials in the setting of graft versus host disease, autoimmunity and kidney and liver transplantation using Tregs have shown the feasibility and safety of this strategy. This review will summarise current knowledge of the latest clinical therapies for CAV and pre-clinical evidence in support of Treg therapy for CAV. We will also discuss the different Treg sources and the considerations of translating this into a feasible immunotherapy in clinical practice in the paediatric population.

## Introduction

Paediatric heart transplant is an established treatment for end stage heart failure in children with congenital heart disease and cardiomyopathy. Though this can significantly improve quality of life, heart transplant recipients will need lifelong medical input to manage the risk of rejection. Cardiac Allograft Vasculopathy (CAV) is a form of chronic allograft rejection and is a critical predictor of long-term survival with nearly half of all paediatric transplant recipients having developed CAV 15 years post-transplant (1). Current accepted management includes immunosuppressive drugs to target the underlying immunological pathophysiology and also medications to target the traditional cardiac risk factors, address inflammation and immune mediated graft injury. There are some interventional options to address severe stenoses (2) however once established, there is no definitive treatment for CAV except for re-transplantation which is not a straightforward solution. There is a pressing need to find a treatment for CAV or prevent its onset in patients receiving heart transplantation.

In the face of major challenges for all solid organ transplant patients such as immune mediated graft rejection and long term side effects and toxicity of immunosuppressive regimes, research has focused on novel immunological approaches that can lead to permanent acceptance of the allograft using regulatory T cells (Tregs) (3). In pre-clinical and early phase clinical trials, we and others have demonstrated promising results with Tregs in patients with solid organ transplants and autoimmunity with feasibility and safety of this strategy and in some cases a hint of efficacy was observed (4). To our knowledge, we are one of the two groups that have established a GMP compatible protocol (5, 6) for the clinical use of Tregs in the setting of paediatric heart transplant for preventing the onset of immune rejection. Our group is focusing specifically on using Treg therapy to target development of CAV. As described in detail below, animal models of heart and vessel transplants support the use of Tregs in the prevention of CAV. However, producing a bespoke immunotherapy that is attainable in a Good Manufacturing Practice (GMP) has its challenges as most of the current clinical research is primarily in adults (4) where obtaining larger volumes of blood from which to isolate Tregs is more feasible than doing so in smaller children.

In this article, we seek to provide a perspective on supporting the use of adoptive therapy with Tregs as immune modulators and as a potential preventative therapy for CAV in children post-heart transplantation. We will reference data from pre-clinical studies which provide a robust foundation for the role of Tregs to prevent solid organ graft rejection and influence the pathophysiology underlying CAV. We will describe methods used so far to isolate Tregs from the blood and we will extend our analysis to Tregs prepared from other sources such as the thymus. We will then set out the practicalities of expanding them in the laboratory to translate to a high-quality autologous cell product that can be administered to adult and in particular to paediatric recipients of heart transplants at the bedside.

## Immunosuppression for heart transplantation: a double edged sword

International Society of Heart Transplant (ISHLT) registry data shows that rejection continues to be a major cause of morbidity and mortality in children post-transplant with early rejection being associated with decrease in overall survival (7). Review of the Paediatric Heart Transplant Study (PHTS) database with data from multiple institutions by Gossett et al, showed that between January 1993 and December 2005 the incidence of early rejection episodes (within the first year of transplant) declined from approximately 60% to 40% (p<0.001); although the incidence of death due to rejection did not change over this time (8). Rejection as defined by the PHTS is an event leading to augmentation of immunosuppressants due to suspicion based on clinical features such as echocardiographic findings or endomyocardial biopsies (8). Over the data collection period, use of tacrolimus, mycophenolate mofetil and azathioprine increased with reduction in choice of cyclosporine as an agent. Multiple factors could have explained this finding of reduced incidence of early rejection. For example, in the later era physicians were less likely to augment immunosuppression based on clinical suspicion alone, but instead place a greater reliance on pathological evidence of rejection seen on endomyocardial biopsies. It could also be hypothesised that different choices of immunosuppressive regimes have contributed to this finding, though this study (8) is not powered adequately to support this. Currently the most common immunosuppressant regime at discharge includes an antiproliferative agent (Mycophenolate mofetil -MMF), a calcineurin inhibitor (Tacrolimus) and oral steroids (methylprednisolone) (9). Despite the desired effects, these agents also have adverse effects of leaving the patient at risk of infection, malignancy and contributing to metabolic effects such as diabetes, hypertension and renal dysfunction (10).

## Cardiac Allograft Vasculopathy (CAV) in the transplant recipient

**Figure 1:**
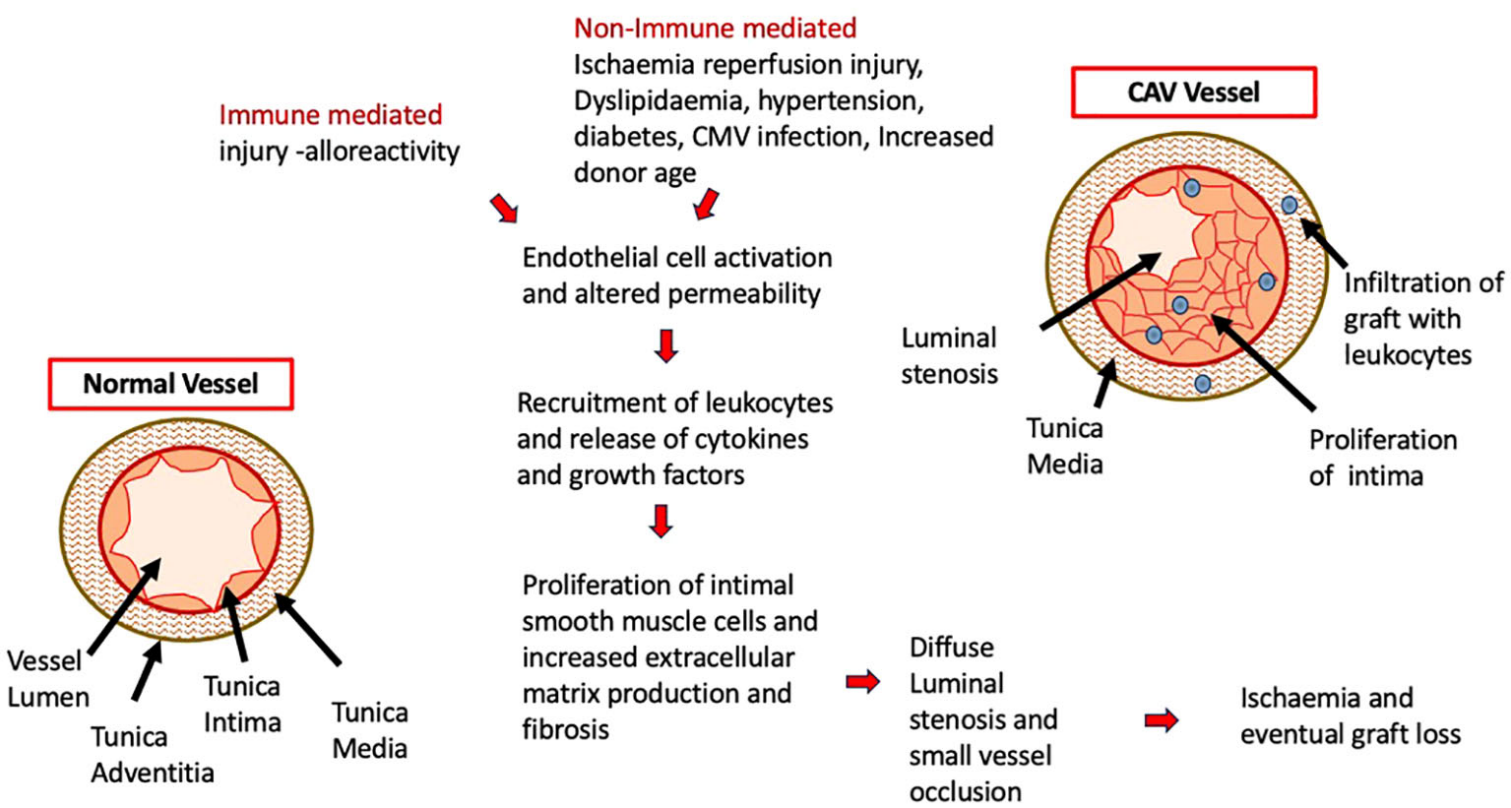
Underlying Pathophysiology of CAV with the cascading effect of initial endothelial cell injury and activation of immune cells. Immune and non-immune mediated factors contribute to this process with the resulting diseased vessel becoming occluded and resulting in graft ischaemia and loss.

CAV is a leading cause of death beyond 3 years after heart transplant and remains an important limitation to long term survival and graft longevity (11). CAV results from a complex interaction between multiple immune mediated factors outlined in **Figure 1**, such as histocompatibility mismatch between the donor and recipient, which triggers an endothelial injury and is propagated by non-immune factors such as ischaemic-reperfusion injury, cytomegalovirus infection, dyslipidaemia, diabetes and hypertension. Endothelial cell activation propagates inflammation by upregulating adhesion molecules and releasing cytokines which draws in immune cells (neutrophils, NK cells, T effector cells) to the site of injury (12). Subsequent cascade of immunological reactions, activation of complement, and cytokine production leads to migration and proliferation of smooth muscle cells into the intima and eventual laying down of extracellular matrix which thickens the intimal layer (13–15). Abnormalities within the intramyocardial microvasculature causes diffuse luminal stenosis within the arteries that supply the allograft, which leads to myocardial ischaemic injury and dysfunction (12). Due to denervation of the allograft following transplant surgery, underlying ischaemia is not always felt or reported by patients, so first clinical presentation and diagnosis may only be made in advanced disease or after graft loss (13). ISHLT data from 2017 reports that by angiography 67% patients (all ages) are free from CAV 10 years post-transplant (7). This has not significantly changed when compared with ISHLT data from 2010 which showed 66% of patients were free from CAV at 10 years (16). It is well known that coronary angiography is not the most sensitive method of diagnosis (13) so early onset disease or true burden of disease may be well underestimated. Following established CAV, graft survival is also significantly reduced across all age groups with infants being worst affected with a median survival of 2 years (7) after diagnosis.

Evidence from recent registered clinical studies, primarily undertaken in the adult recipients of heart transplants demonstrates some of the therapeutic strategies for CAV. This is summarised in **Table 1**. As CAV represents a chronic allograft rejection process and patients with increased frequency of early rejection episodes within a year of transplant are at greater risk of CAV (17), an important focus for clinicians managing heart transplant recipients is the choice of induction and maintenance immunosuppression agent. In fact the ISHLT 2016 report of paediatric heart transplants undertaken between 1994 and 2014 reported a small, but statistically significant higher CAV free survival (86% at 5 years and 68% at 10 years post-transplant) in patients that had induction therapy compared with those that did not have induction therapy (85% at 5 years and 65% at 10 years) (17). With regards to maintenance immunotherapy, MMF has shown superiority to azathioprine in combination with a calcineurin inhibitor with 70% being free of CAV at 5 years compared to 47% in adults heart transplant recipients taking ciclosporin and azathioprine (18). This is likely due to the antiproliferative action of MMF that targets smooth muscle cells and fibroblasts which contribute to intimal lining thickening in CAV (2). Newer agents such as Proliferation Signal Inhibitors (PSI) such as Everolimus have shown promise in studies at preservation of the coronary artery lumen and reduced CAV incidence in patients when compared with azathioprine (19). More recently SCHEDULE *(NCT01266148)* trial (20) with *de novo* Everolimus initiation with early cyclosporine withdrawal showed significantly reduced CAV progression at 12 months compared with the conventional ciclosporin. TEAMMATE trial *(NCT03386539)* is the first multicentre randomised controlled trial (21) in paediatric heart transplants recipients to investigate if a combination of Everolimus and low dose Tacrolimus is associated with a lower total burden of transplant complications (kidney disease, CAV and CMV) compared with Tacrolimus and MMF (combination which is a commonly practised dual therapy in paediatrics). Preliminary results presented at recent scientific meeting in November 2023 has shown numerically lower CAV and renal dysfunction in the Everolimus group with higher report of infection and lymphoproliferative disorder however it is unclear if all of these findings are statistically significant. The authors have confirmed that Everolimus treatment in combination with low dose tacrolimus is safe to initiate in children 6-months post-transplant (22). Full results from this trial are eagerly awaited. Other well established treatment strategies for CAV include managing the traditional cardiovascular risk factors with antiplatelets (NCT04770012), antihypertensives (NCT01078363) and hyperlipidaemia. A number of novel lipid lower agents (as listed in **Table 1**) in addition to statins such as Evolocumab (NCT03944577) or Alirocumab (NCT04193306) which are Proprotein Convertase Subtilisin Kexin type 9 (PCSK9) inhibitors are also being tried recently. In terms of interventional approaches, coronary revascularisation procedures have been explored but have not been shown to provide survival benefit (2, 13) because the concentric, progressive and diffuse nature of CAV in distal vessels makes transplant patients non-ideal candidates with an important risk of re-stenosis, however novel drug eluting stent options are being investigated (NCT02377648).

**Table 1:** Summary of recently registered clinical trials for CAV.

Study ID	Location	Start date (Year)	Study Design	Enrolment (Number of patients)/Age	Proposed Intervention/ Treatment target	Status
NCT05373108	Los Angeles	May 2022	Single centre. Intervention on single cohort, unblinded, No control group	19/ >18 years	Oral endothelin receptor antagonist (Macitentan)	Completed
NCT01157949	Los Angeles	Nov 2010	Single centre. Randomised with treatment and control arm. Unblinded.	0/ 18-70years	Thymoglobulin infusion post-transplant	Withdrawn (No approval)
NCT05485467	Ljubljana	June 2022	Single centre. Prospective observational cohort study	55/ >18 years	Measuring CD34+ cells in peripheral blood in patients post-transplant with and without CAV	Completed
NCT03944577	Nebraska	July 2019	Single centre. Intervention in single cohort. Unblinded. No control group	26/19-80 years	Evolocumab (PCSK9 inhibitor) given to cohort with existing CAV.	Completed
NCT04770012	Ottawa	June 2021	Multiple centre Randomised to one of either treatment vs placebo. Triple blinded	135/ >18 years	AERIAL Antiplatelet drug in heart transplant recipients post-surgery Aspirin vs Clopidogrel vs placebo	Recruiting
NCT01278745	USA	Sept 2011	Multicentre Randomised	362/ 18-75 years	Rituximab with standard immunosuppression vs placebo with standard immunosuppression	Terminated (inability to meet accrual goals in funding period)
NCT02082821	Boston	Jan 2014	Single centre Observational cohort study	200/18-65 years	Blood tests for genetics (loss of function mutation P2X7R gene) and development of CAV in heart transplant recipients with a view to producing a targeted gene therapy drug	Completed
NCT01266148	Scandinavia	Nov 2009	Multicentre, randomised to treatment group or standard care group. Open label	115/ 18-70 years	Intervention group- Everolimus with complete cyclosporine withdrawal 7 to 11 weeks after heart transplantation or standard cyclosporine-based immunosuppression	Completed
NCT02377648	Verona	Jan 2015	Single centre, Intervention group alone, no control. Open label (unblinded)	34/ >18 years	Everolimus-Eluting Bioresorbable Vascular Scaffold (ABSORB) used in presence of CAV associated angiographic lesion with symptoms of ischaemia.	Completed
NCT04226521	Zagreb	Jan 2018	Single centre, randomised to intervention and standard of care immunosuppression vs standard of care alone	30/ 18-65 years	Extracorporeal photopheresis treatment (10 sessions) given to randomised patients who are receiving usual immunosuppression and monitored for CAV and adverse effects	Unknown status
NCT04193306	Prague	Nov 2019	Single centre, randomised to treatment or placebo. Double blinded.	126/ >18 years	Alirocumab (PCSK9 inhibitor) given to heart tx recipients in addition to standard statin therapy	Recruiting
NCT01078363	California	June 2009	Single centre. Randomised to treatment or placebo Double blinded	96/ >12 years	After baseline investigations for CAV, participants are randomised to ramipril vs placebo groups	Completed
NCT06147271	Brazil	Nov 2023	Single centre standard care to all patients with some randomised to intervention group or no intervention (control)	80/ >18 years	Efficacy and safety of adding SGL2 inhibitors eg dapagliflozin or empagliflozin conventional post-Tx treatment compared with the treatment of isolated conventional care	Not yet recruiting
NCT01186250	California	July 2010	Single centre, randomised to group receiving intervention vs placebo. Blinded	18/ >18 years	Pioglitazone vs placebo in heart transplant recipients	Completed
NCT00966836	Bologna	April 2009	Single centre Randomised to study effect of pre-emptive anti-CMV treatment with universal anti-CMV prophylaxis on CMV infection. Patients will be additionally randomized to receive either MMF or Everolimus. Open label	100/ >18 years	PROTECT 1.Everolimus + pre-emptive valganciclovir 2. MMF + prophylaxis valganciclovir 3. Everolimus + prophylaxis valganciclovir 4. MMF + pre-emptive valganciclovir Note- Pre-emptive valganciclovir given only if PCR positive. All patients have usual immunosuppression in addition (CsA and prednisolone)	Unknown status
NCT03386539	Boston	Jan 2018	Multi centre. Randomised to two groups of treatment Open label	211/ <21 years	TEAMMATE Everolimus + low dose tacrolimus vs tacrolimus + MMF (active comparator)	Active, not recruiting. Expected completion April 2024

Even with the potential benefits of PSIs or the aspiration to find the ideal combination of immunosuppressant drugs to reduce incidence of CAV and increase the longevity of the graft; the side effect profile of immunosuppressant inhibitors is not to be underestimated. This includes renal toxicity, hyperlipidaemia, hypertension, increased risk of infections and impaired wound healing (10). Especially as some of them are traditional cardiac risk factors themselves, that contribute to the non-immune factors implicated in the pathophysiology of CAV. Overall, there is no specific intervention that is proven to prevent or reverse pathophysiology once CAV has been established (2). The only definitive treatment of CAV is re-transplantation which is an enormous undertaking but also limited by availability of donor organs.

In summary, there is an unmet need to find a solution to prevent the onset of CAV which can have a significant impact on long term survival of paediatric childhood recipients. Furthermore, finding an alternative or therapy with the potential to reduce the need for traditional immunosuppressant and establish operational tolerance will also improve the short to medium term quality of life of these children. One such novel therapy proposed is the use of autologous regulatory T cell (Tregs) therapy which has shown much promise in animal and early clinical studies.

## The effect of regulatory T cells on immune cells

CD4^+^ Tregs are a subset of T cells characterised by constitutive expression of IL-2 receptor alpha chain (CD25), low expression of IL-7 receptor alpha (CD 127) and expression of transcription factor Forkhead box P3 (FOXP3). Broadly, there are two types of Tregs; those that have been generated in the thymus (thymus- derived Treg, tTreg) and a subset of CD4+ T cells that are FOXP3 negative but under the right circumstance differentiate and become FOXP3 positive *in vivo* and these are known as ‘peripherally induced’ T regs (pTreg) (23). Both subsets are present within the peripheral blood compartment however discriminating between these two cell populations can be challenging due to the lack of specific cell markers (24, 25).

**Figure 2:**
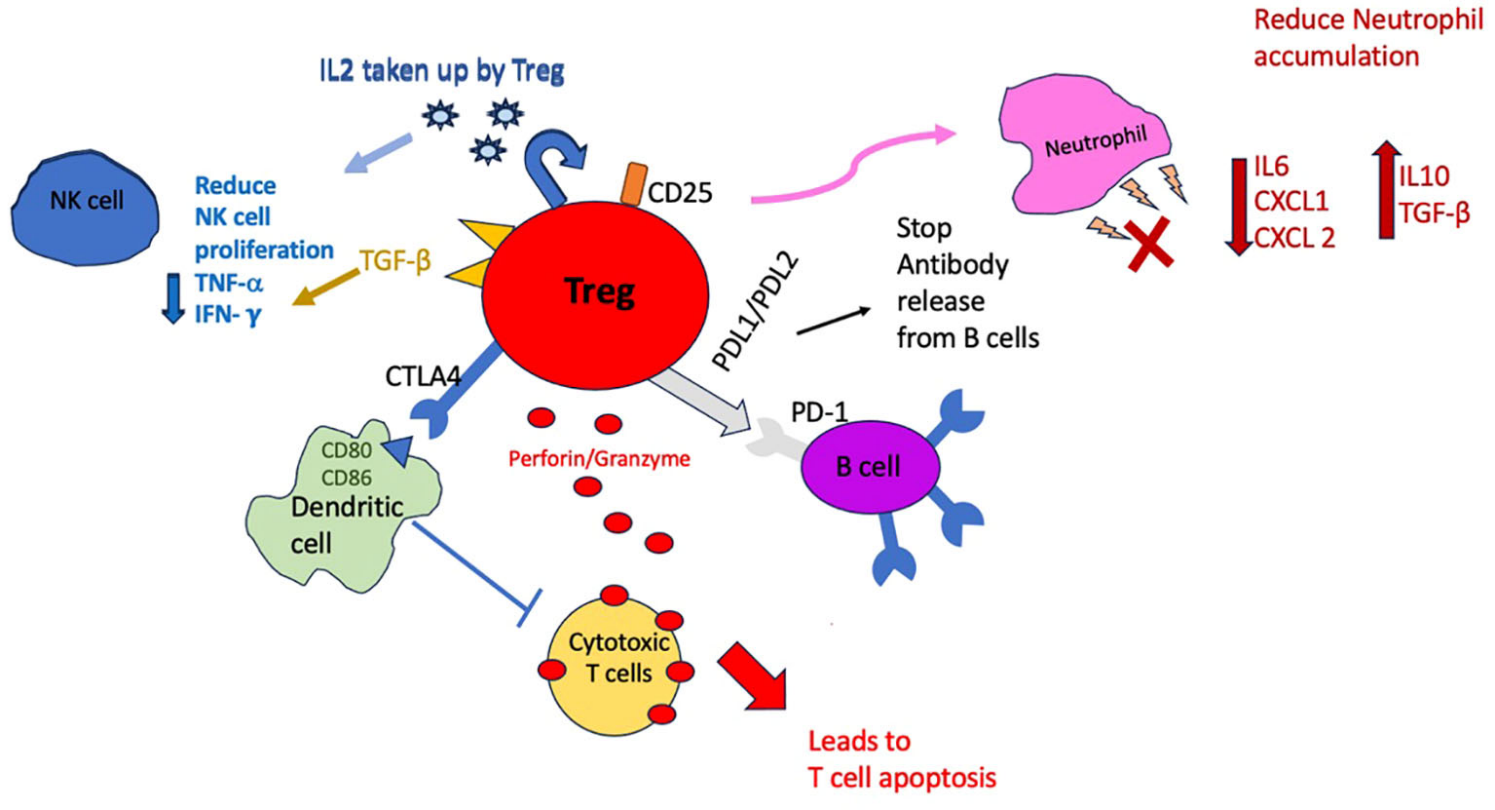
Schematic diagram to demonstrate the different ways in which Tregs can suppress immune cells. Cell to cell contact mechanisms include producing perforin/granzyme and directly influencing dendritic cells and B cells with surface receptors but also directly influencing neutrophils by reducing expression of chemoattractants. Indirect mechanisms include uptake of IL-2 by Tregs and depleting the microenvironment which in turn will reduce NK cell and effector cells proliferation.

Tregs can act on different immune cells directly (cell to cell contact) or via soluble factors as outlined in **Figure 2**. Tregs can interact with dendritic cells (DCs) via CTLA-4 and downregulate co-stimulatory molecules inhibiting T effector cells activation (4, 26). Tregs can also directly suppress antigen specific B cells to stop them releasing antibodies to specific antigens via inhibitory molecules such as PD-1 and 2 ligands (4). Via cell to cell contact, Tregs by producing perforin and granzyme can kill cytotoxic T lymphocytes (27); this mechanism of action of Tregs is of relevance in CAV, as cytotoxic (CD8+) T lymphocytes are implicated in infiltrating the graft and contributing to ‘lymphocytic endothelialitis’ which is characterised by immune cells accumulation within the subendothelial space of the vessel causing it to swell (28, 29). Tregs are scavengers of IL2 and by depleting the microenvironment of this essential cytokine, can hamper proliferation of NK cells as well as other effector cells. In addition, Tregs can also express Transforming Growth Factor- beta (TGF-β) which can directly downregulate NK cells activity and reduce their proliferation. The control of NK cells by Tregs is also important in CAV as they are implicated in early development of CAV (30) and can attract other inflammatory cells by production of interferon gamma and Tumour Necrosis Factor-alpha (TNF-α). Tregs can also influence the innate immune system by reducing the accumulation of neutrophils by affecting their ability to produce chemoattractants (CXCL1/CXCL 2) and also inducing them to be less inflammatory by encouraging production of TGF-β and to make less IL6 (31).

The mechanisms by which Tregs are capable of suppressing immune cells have been dissected in pre-clinical models of autoimmune disease (25), graft versus host disease (32), solid organ transplantation (33) and CAV (30, 34–38). These studies provide robust proof of concept that can be built on with clinical studies involving human patients.

## Evidence for use of Tregs in pre-clinical models of CAV

Murine models studying CAV generally require cardiac transplantation which is undertaken between mice ‘heterotopically’. Vascularised cardiac allografts from donor mice are transplanted within the abdominal cavity of recipient mice using various methods similar to the method described in Corry et al. (39). The aorta and pulmonary artery of the donor graft is anastomosed end-to-side to the recipient’s aorta and vena cava. Rather than the whole heart grafts being transplanted, Warnecke et al. transplanted isolated aorta grafts from donors into recipient (34). Mice are selected to provide various combinations of histocompatibility in relation to MHC antigens to study mismatch. Some mice may be genetically engineered to be fully immunodeficient or deficient in specific cell lines and exposed to allogenic blood cells to mimic solid organ mismatch. Humanised mouse models (37, 40) involve transplanting immunodeficient mice with human arterial segments (such as side branches of internal mammary arteries) that have been collected from human bypass surgeries. Mice are then reconstituted with allogenic peripheral blood cells that are HLA mismatch to donor vessels to mimic a model of CAV. The grafts are harvested at specified time points after exposure and histopathology or immunohistochemistry techniques are conducted to monitor the nature and severity of any rejection.

Summary of data from the studies in **Table 2** support that CAV is a chronic rejection process mediated by a number of immune cells that can infiltrate the graft including CD8+ T cells, NK cells and CD4+ cells (30, 41) and are responsible for causing neointimal thickening and eventual luminal stenosis that leads to graft ischaemia and damage. Regulatory T cells are involved in ameliorating the development of CAV, as adoptive transfer of these to recipients mice of transplant conferred protection of the graft from CAV (34, 37, 38, 40, 42) and improve graft survival in some studies (35, 36, 41). Several studies from above that have investigated the underlying mechanism show that one of the ways that Tregs suppress is by directly affecting T effectors (34), NK cells (30) and via an IFN-gamma mediated pathway (40). Most of the humanised model studies that test adoptive transfer of Tregs do so by obtaining Tregs via a purification process from peripheral blood of the donor or the recipient of the allograft and expanding them polyclonally with general activation (38, 40–42). Tregs obtained and activated in this manner are not specifically selected to recognise a single peptide. Though it is thought that Tregs are more likely to recognise self-antigens, they have the capacity to also recognise a broad range of non-self antigens (43). We have investigated the expansion of donor-specific Tregs and the results in a mouse heterotopic transplant model showed that following adoptive transfer of donor-specific Tregs graft survival was significantly prolonged (35). In addition, myocardial architecture was maintained, and luminal occlusion was inhibited. The same result was obtained in the humanised mouse of human vessel transplant when human polyclonal Tregs were injected (38). Use of agents such as rapamycin together with Treg transfer were seen to have an additive effect at reducing CAV in the same humanised murine model of vessel transplants (37).

**Table 2:** Summary of studies that have shown involvement of Tregs in the pathophysiological development of Coronary Allograft Vasculopathy in animal models.

Study	Model of CAV	Description of Study Design and Results Summary
**Warnecke et al., 2007 (** 33)	Murine model	CAV (luminal occlusion of graft vessels due to neointimal formation) developed in immune deficient mice that received T effector cells. This was significantly reduced (P<0.05) in the group that received concurrent adoptive T reg therapy.
**Tsang et al., 2009 (** 34)	• *In vitro* culture of Tregs • Murine model	*In vitro* studies showed that Tregs with indirect specificity (to recognise specific alloantigen peptide) could be generated in addition to Tregs that were self-reactive. Injection of both T reg lines (self-reactive and allospecific) to fully immune competent mice undergoing mismatched heart transplants could prolong graft survival with allospecific Tregs being only slightly more effective that autoreactive Tregs.
**Zhu et al., 2013 (** 35)	Murine model	Mice with gamma-delta TCR deficiency (also those that received anti gamma-delta antibody) had significantly greater survival of allografts compared with wild type mice. Harvest of tissue from mice with gamma-delta TCR deficiency showed significantly less CAV when compared with wild type with evidence of increased number of Tregs (CD25+ FOXP3+) in the graft in the context of reduced expression of IFN gamma, Hmgb1 and IL-17. To further investigate the role Tregs played in CAV, gamma-delta TCR deficient mice were injected with anti CD25 monoclonal antibody (to deplete Tregs) prior to transplantation and the prolonged allograft survival time was abrogated in those mice (P<0.05) compared with non-Treg depleted mice.
**Hirohashi et al., 2014 (** 29)	• *In vitro* studies • Murine model of CAV	Mice that underwent NK cells depletion showed no signs of CAV at 3 weeks post transplantation compared to control group (No NK cell depletion) mice (6 out of 8 allografts showed early CAV changes) suggesting that early phase CAV post-transplant is NK cell mediated. Allografts of mice treated with anti CD25 antibodies (Treg depletion) showed similar frequency of CAV as allografts of control group mice (12/12 vs 6/8). Histology identified that the CAV lesions in T reg depleted mice were more cellular or ‘advanced’ with significantly increased stenosis (82% vs 23%) as compared with untreated mice.
**Sherman et al., 2009 (** 37)	Murine model	Immunodeficient mice were reconstituted with CD4+CD25- T lymphocytes (T effectors) and underwent cardiac transplantation. Tregs were given (before transplantation) in various numbers and compared with control group (no Tregs therapy given). In the control group, donor hearts reproducibly developed CAV within 40 days. Mice given T reg treatment prior to transplant had allografts that showed significantly less (P<0.05) severe intimal lesions.
**Harper et al., 2018 (** 41)	• Human *in vivo* studies • Murine model of CAV	A range of donor T lymphocyte subsets were detectable in human recipients’ blood post-transplant at various time points that are likely released from donor graft (‘passenger’ donor T cells). Treg depletion of recipient mice prior to and after transplantation (with anti-CD25 antibodies) with mismatched mice hearts showed accelerated CAV development in grafts which was likely mediated by passenger donor T cells at the time of graft transplant. When heart donors were from mice that were genetically T cell deficient, these allografts did not trigger recipient response and grafts survived indefinitely with no CAV (even with T reg depletion of recipient) supporting that CAV is likely mediated by donor T cells.
**Hester et al., 2012 (** 36)	Humanised murine model	Arterial segments of human arteries were transplanted into immunodeficient mice and then exposed to allogenic HLA human blood alone (control) or in combination with rapamycin and expanded Treg infusion (vessel donor’s own Tregs) at various doses. CAV was seen in control mice after the graft was harvested with evidence of intimal expansion and luminal occlusion. The addition of rapamycin or T reg therapy to the model reduced the neointimal formation and CAV development. Combination therapy (rapamycin and T reg) significantly reduced development of CAV in the graft.
**Nadig et al., 2020 (** 39)	Humanised murine model *In vitro* expansion of Tregs isolated from peripheral blood donors	Immunodeficient mice underwent transplantation of human arterial segments and exposed to allogenic blood or autologous blood (control). Grafts were harvested to check for CAV. Significant vascular intimal proliferation and luminal narrowing was seen in grafts that were exposed to allogenic blood compared to vessels of mice in the autologous blood group. CAV in the mice given concurrent Treg infusion was significantly less compared to mice given allogenic blood alone. *In vitro* studies suggest that this is through a IFN-gamma pathway as expression of this is diminished with concurrent Treg therapy.
**Ravichandran et al., 2021 (** 40)	Murine model	Allogenic heterotopic transplant between mice with and without concurrent co-stimulatory blockade (to reduce risk of acute rejection) was performed. Mice that had Costimulatory blockade showed fibrosis and significantly increased T, B and NK cells with CAV at 45 days post-transplant. Mice that received additional IL2 therapy showed attenuated levels of graft fibrosis and their grafts had more FOXP3 expressing cells and reduced number of CD8+ and NK cells compared to costimulatory treatment alone. Higher levels of exosomes with cardiac antigens were isolated from mice that had CAV compared to control. Exosomes obtained from mice that had costimulatory +IL 2 treatment showed greater expression of immunoregulatory markers (PD-ligand 1, CD73 and FOXP3) compared with exosomes isolated from mice with CAV (costimulatory only, no IL2 treatment)

## Considerations of translating adoptive Treg cell therapy from the laboratory to the bedside

Adoptive Treg cellular therapy involves producing a bespoke infusion of Tregs that is produced under Good Manufacturing Practice (GMP). Tregs have been isolated from the peripheral blood of the patient with autoimmunity or receiving a solid organ (e.g. kidney or liver) or from the donor of the bone marrow (BM) in BM transplantation. However, an important consideration when producing this novel therapy for patients includes choice of starting material for the Treg infusion and if collection of this material is feasible in the target patient population. The next steps include developing a protocol for isolating and expanding Tregs *in vitro* to obtain the dose for each patient based on weight, product quality testing and monitoring in line with the requirements from the Medicines and Healthcare products Regulatory Agency (MHRA) and finally recruitment of patients in the clinical trial after all the necessary ethical approvals are in place.

The first clinical trials with Tregs published were in Bone Marrow Transplant (BMT) patients in which Tregs were generated from the BM donor (44–46). The success of these first few clinical trials paved the way for additional clinical trials not only in BMT patients but also in patients receiving solid organ transplants. Todo et al. infused a Treg enriched product that was generated by culturing PBMC with donor derived cells in the presence of antibodies inhibiting co-stimulatory molecules. Ten patients who underwent liver transplants and splenectomy were treated with the Tregs (47). They were able to wean 7 patients off immunosuppression therapy after Treg cell infusion however 3 patients had rejection (these 3 patients had autoimmune hepatitis), so oral conventional immunotherapy was restarted for them and the trial was stopped early. Altogether, they demonstrated safety following infusion of their Treg containing product and for seven patients they demonstrated that operational tolerance can be actively achieved after liver transplantation.

During the same period, a few groups including ours have used Tregs purified from the blood of transplant recipient and expanded either polyclonally or in a donor-specific manner. Tregs have been infused into patients post kidney and liver transplantation [reviewed in Romano et al., 2019 (4)]. These trials used good manufacturing practice (GMP) protocols for the generation of the Treg product via leukapheresis of peripheral blood of patients. We have completed two clinical trials (48, 49) in renal (The ONE Study) and liver (ThRIL) transplant patients. The Tregs were purified either from large volume of blood or from leukapheresis and then expanded *in vitro* for 24-36 days as the doses per patients used ranged from 1 to 10x10^6^/Kg. We have demonstrated that Treg therapy with polyclonal Tregs is feasible, safe and we showed some biological effect of the Tregs. More recently, we have started the TWO study (50) a phase 2b randomised controlled trial of Treg therapy (similar product to the ONE study) in kidney transplant patients. As previously, the generation of the Treg product requires a starting blood draw from the patient exceeding 350ml as stated in the protocol (43). This is simply not feasible from a clinical perspective in the paediatric population so alternative sources of Tregs have had to be investigated.

## The thymus as alternative source to generate the Treg product

We and others have investigated alternative sources of starting material for Treg production such as thymus tissue. An added advantage of using a thymus as a viable source in the paediatric cardiology cohort is that this may be routinely removed during paediatric congenital cardiac surgery (including during heart transplant surgery) in order to improve the field of view of the surgeons. This tissue is often discarded however retaining it, is an added advantage as it is where the Tregs originate. In an elegant study, Dijke et al. isolated and characterised Tregs from thymi of children removed during routine cardiac surgery and compared with Tregs isolated from peripheral blood of healthy adult donors. They found that the yield of Tregs from 1g of thymus tissue contains 500 times the amount of Tregs as 1ml of blood from adult donors and once expanded, thymic Tregs had stable FOXP3 expression and were capable of suppressing allogeneic T cells *in vitro* more potently compared with blood derived Tregs (51).

After obtaining this starting material, thymus tissue needs to undergo mechanical or enzymatic digestion (6, 50). The thymocytes will then undergo an enrichment process to purify the Tregs. Isolated Tregs are expanded in culture polyclonally (52) in the presence of IL2 (53, 54) and Rapamycin. Samples of the resulting Treg product will undergo rigorous quality control checks using flow cytometry to check expression of various markers including FOXP3 (phenotype check) as well as suppression capability. The final product can then be injected fresh, or it will be frozen down until use according to the trial. Additional testing for sterility, phenotype and stability assays after cryopreservation will also take place prior to infusion to patients.

Another consideration of Tregs based cellular therapy is their heterogeneity and ‘plasticity’ which is the variability of Treg subpopulations and also their ability to demonstrate features of immune cells that they influence (4). Subpopulations of Tregs in circulation can be naïve/resting (CD45RA^+^FOXP3^low^), effector type (CD45RA^-^FOXP3^high^) or cytokine producing (CD45RA^-^FOXP3^low^) depending on level of FOXP3 or CD45RA expression (55). Multiple rounds of polyclonal stimulation during the production phase, and also exposure to inflammatory cytokines can push circulating Tregs into becoming cells that release inflammatory cytokines like IL 17 and interferon gamma themselves (56). Furthermore, in order to be effective as suppressors, Tregs must retain stability in a pro-inflammatory environment which may well be in the setting of a transplant patient receiving a T reg infusion. Dijke et al. showed that thymic derived Tregs consistently remain stable and suppressive even in inflammatory conditions in contrast to blood derived/peripheral Tregs that are more unpredictable in their response (51). Furthermore, thymic derived Tregs have a substantial proportion of naïve (CD45RA^+^) Tregs that have not been exposed to differentiation and are more stable compared to CD45RA^-^ subpopulation seen in greater proportion of peripheral blood derived Tregs from adult populations (55). In 2021, we published a GMP compatible protocol (6) using thymic derived Tregs from children which reproduced findings noted by Dijke et al. We compared total thymic Tregs and the subpopulation of CD45RA^+^ Tregs (after cell sorting) obtained from the thymus of 11 paediatric patients which were then expanded for potential clinical application. These cells showed high level of suppressive capacity, stability and retained these features after freezing and thawing supporting translation into the clinic.

## Thymic Treg therapy and the heart transplant recipient

The first in human thymic derived Treg infusion into a paediatric heart transplant recipient was reported by a group in Spain in 2023 (5). They developed and published their own GMP compatible protocol (57) and following approval from their medicine regulatory body, commenced recruitment into a phase 1/2 clinical trial with autologous Treg therapy in children post heart transplant to prevent rejection. Tregs were generated from thymus tissue removed at the time of transplant and following a brief ex vivo expansion, the cells were infused fresh back to the patient 9 days post-transplant. They also included a control cohort of 6 children (who did not receive a Treg infusion) but underwent the standard treatment of thymectomy and heart transplant. All children had regular immune monitoring at various time points for up to 2 years post-transplant. In the control group, peripheral Treg levels transiently increased within the first few weeks of transplant and then showed a progressive decline from 9 months post-transplant. In contrast, their trial patient had an increase in Treg frequency in blood after the infusion and also maintained T reg values higher than pre-transplant levels throughout the 2 year follow up period. Control group data is in keeping with a cohort study published by the same group in 2021 which aimed to follow heart transplant patients (n=7) undergoing thymectomy and standard immunosuppression after heart transplant to identify dysregulation of T cells compartment in peripheral blood (58). This cohort study showed a significant decline in the peripheral T reg levels 7 months post-transplant with a significant increase in effector T cells coinciding with the T reg drop. The prospect of boosting the Treg pool in the child who had the autologous T cell therapy 2 years later is remarkable, as in comparison we demonstrated that Tregs given in adult patients post liver transplants were detectable in their blood for only 1 month after (49). One may hypothesise that this persistence in circulation months after infusion could be linked to the richer ‘naïve/resting’ subpopulation of Tregs that are isolated from the thymus compared to those isolated from peripheral blood or something to do with their inherent nature or metabolism that will require further study.

## Targeting the allograft with Treg cell therapy

The clinical trial in Spain showed feasibility of generation of autologous Treg therapy at a dose of 20 x 10^6 cells/kg with no reported toxicity from the infusion. However limited conclusions about the effect can be drawn with results from a single patient. Furthermore, though they demonstrated boosting of the peripheral Treg pool in the treated patient, no definitive biopsy data to show Treg infiltration of the graft was demonstrated. Additionally, obtaining further data on the subtype of circulating Tregs after infusion would be useful. Specifically applying this treatment to minimise the onset of CAV; an important question to consider is if the Tregs infused as part of therapy are able to make their way to the allograft, particularly if they are polyclonally expanded and not primed to recognise a specific donor heart alloantigen. This is relevant because if Tregs are not able to navigate to the graft to modulate chronic inflammation as seen in patients with CAV, one might have to consider generating donor specific Tregs or Tregs with chimeric antigen receptor technology (CAR-Tregs) to direct them to the donor graft directly. This will no doubt add further steps and greater complexity to the production of the Treg therapy. However, there is some scientific evidence that recipient circulating Tregs can accumulate within an allograft. Schmidt-Lucke et al. demonstrated this in an elegant study in adult heart transplant recipients by measuring number of Tregs within cell populations from samples taken from the aortic root and coronary sinus to obtain a ‘transcoronary gradient’ (59). The control group were non transplanted patients with a diagnosis of coronary artery disease who were undergoing routine cardiac catheter and angiography procedure. Only in the heart transplant recipients, Tregs decrease after passage through the coronary arteries (p<0.05) suggesting that they were being recruited into the graft. Furthermore, FOXP3 staining was undertaken on myocardial biopsies which confirmed presence of FOXP3 positive cells (i.e. Tregs) within the allograft. In terms of peripheral Treg pool, heart transplant recipients had significantly lower circulating Tregs by absolute numbers but also as proportion of lymphocytes compared with control patients (healthy patients with no known heart disease). The authors speculate that this may be due to the immunosuppressive regime or could also be due to the active migration and recruitment of Tregs into the allograft (59). Further study is required to assess the effects of immunosuppressive medication and also induction therapy (such as Basiliximab or anti-thymocyte globulin) utilised in paediatric heart transplant patients on circulating Tregs both *in vivo* and *in vitro*, as it will help establish the optimal timing of delivering the autologous T reg cell therapy for future clinical trials.

## Future directions and conclusions

In summary there is encouraging pre-clinical and clinical data for Tregs to influence host immune response to heart allografts and to act in particular to regulate the onset of CAV. This strategy holds promise as a novel treatment to prevent the development of CAV and prolong the life of the transplanted organ. Furthermore, weaning immunosuppressive drugs required can limit their undesired side effects and improve quality of these young patients’ lives.

There are several clinical trials already that have shown that generating autologous T reg therapy for patients is technically feasible and safe to administer with one previous trial focusing on infant recipients of heart transplantation. We are planning to undertake a Phase 1 trial to formally establish the feasibility and safety of administering autologous Treg therapy in nine paediatric heart transplants recipients to prevent the onset of CAV. Our aim is to collect evidence from immune monitoring and histology from biopsies to support translating this novel cell therapy from the laboratory to the bedside. If our clinical trial demonstrates success in this early phase in paediatric heart transplantation, it will establish a precedent for cellular, Treg, immune therapy in children to become part of standard care in children receiving heart transplants.
